# Impact of Refractive Surgery on Visual Outcomes and Patient Satisfaction: A Six-Month Assessment in Palestine

**DOI:** 10.7759/cureus.95198

**Published:** 2025-10-22

**Authors:** Ithar M Beshtawi, Mohammad Shehadeh, Mohammed Aljarousha, Eman Keelani, Mona Sinan

**Affiliations:** 1 Optometry, Faculty of Medicine and Allied Medical Sciences, An-Najah National University, Nablus, PSE; 2 Ophthalmology, An-Nour Eye Center, Nablus, PSE; 3 Optometry, Faculty of Health and Life Sciences, Management and Science University, Shah Alam, MYS; 4 Optometry, An-Nour Eye Center, Nablus, PSE

**Keywords:** activities of daily living, eye health equity, patient satisfaction, quality of life, refractive surgery, vision impairment, visual outcomes, well-being

## Abstract

Background: Refractive surgery has advanced significantly recently, leading to improved visual outcomes and increased patient satisfaction. This study aimed to assess vision quality, satisfaction, happiness, ability to perform daily activities, and postoperative symptoms among patients who underwent refractive surgery in Palestine.

Materials and methods: This prospective observational study included 100 patients (69 females; mean age: 28.7±6.7) who underwent refractive surgery. Visual acuity and the spherical equivalent (SE) of manifest refraction were measured preoperatively and six months postoperatively. Patient satisfaction was evaluated via structured telephone interviews using a validated questionnaire covering demographics, motivations for surgery, satisfaction and happiness, and postoperative symptoms.

Results: 86% of participants achieved uncorrected visual acuity (UCVA) between 0.0 and -0.2 logarithm of the minimum angle of resolution (logMAR), with a mean postoperative SE of -0.2±0.3D. A significant correlation was found between preoperative SE and postoperative UCVA (r=0.4; p=0.0), as well as between preoperative best-corrected visual acuity (BCVA) and postoperative UCVA (r=0.4; p=0.0). High levels of satisfaction (84%; mean score 3.8±0.3), happiness (90%; 3.9±0.3), goal achievement (93%; 3.9±0.2), improved quality of life (93%; 3.9±0.2), and willingness to recommend the procedure to others (96%; 3.9±0.2) were reported. Overall satisfaction was strongly correlated with both preoperative SE (r=0.8; p<0.0) and postoperative UCVA (r=0.6; p<0.0). Between 56% and 95% of participants reported improved ability to perform daily activities, which significantly correlated with overall satisfaction and happiness (p≤0.05). Less than 10% reported adverse symptoms, which didn't correlate with postoperative SE or UCVA (p>0.05).

Conclusions: Refractive surgery would significantly improve vision, quality of life, and the ability to perform daily activities. The majority of patients were satisfied, pleased with the outcomes, and willing to recommend the surgery to others.

## Introduction

Refractive errors are highly prevalent worldwide and can lead to vision impairment or even blindness if left uncorrected [1]. Such impairments significantly affect the individuals' ability to function independently, highlighting the importance of addressing refractive error as a key step towards achieving health equity. Various options exist for vision correction, including spectacles, contact lenses, and refractive surgical procedures, all of which aim to alleviate the challenges in daily activities caused by blurred vision [2].

Recent advances in refractive surgery techniques have contributed to the growing popularity of these procedures as alternatives to spectacles and contact lenses [3]. This increased demand is attributed to several advantages, including rapid visual recovery, stable visual outcomes, improved cosmetic appearance, low complication rates, minimal postoperative discomfort, and enhanced patient satisfaction and quality of life (QoL) [4-7].

These benefits align closely with the United Nations Sustainable Development Goal 3 (SDG 3) (Good Health and Well-Being). Assessing patient satisfaction and postoperative well-being is therefore essential, not only for clinical evaluation but also for improving patients' mental and physical health outcomes [8]. Prior studies have demonstrated substantial improvement in visual performance, including uncorrected visual acuity (UCVA), refractive accuracy, and contrast sensitivity, following refractive surgeries [5,6,9]. These improvements have also been observed in individuals with high myopia [6] and in those with combined high myopia and presbyopia [10]. Such enhancement in vision quality contributes to broader global health goals, including SDG 10 (Reduced Inequalities in Healthcare Access), particularly by expanding equitable access to eye care in low-resource settings.

However, patient satisfaction can vary across populations due to cultural factors, socioeconomic conditions, and differences in healthcare systems. In Palestine, commonly performed refractive procedures include laser in situ keratomileusis (LASIK), photorefractive keratectomy (PRK), and femtosecond-assisted (Femto) small-incision lenticule extraction (SMILE). To date, no previous studies have assessed patient satisfaction following refractive surgery within the Palestinian context. This presents a critical gap in locally relevant health data needed for evidence-based healthcare planning and policy-making.

This study aimed to evaluate patients' motivations for undergoing refractive surgery, their satisfaction and happiness with the outcomes, their satisfaction with daily activities, and any adverse postoperative symptoms. These findings will help clarify both the clinical and psychosocial impacts of refractive surgery among Palestinian patients while also establishing a benchmark for patient satisfaction that can guide the development of culturally and economically appropriate refractive services in Palestine. Ultimately, this research supports the advancement of a resilient and inclusive healthcare system in alignment with SDG 3 (Good Health and Well-Being), SDG 9 (Industry, Innovation, and Infrastructure), and SDG 10 (Reduced Inequalities in Healthcare Access).

## Materials and methods

This prospective observational study included patients who underwent refractive surgery (PRK, LASIK, or Femto SMILE) at multiple centers serving patients from across Palestine. All surgeries were performed by a single experienced ophthalmologist, following standardized surgical protocols [3] to ensure consistency in outcomes. For LASIK and PRK procedures, a modern excimer laser system calibrated according to manufacturer specifications was used to ensure precise ablation. Femto SMILE surgeries were performed using a state-of-the-art femtosecond laser with settings optimized for safety and efficacy. Intraoperative events were minimal and managed according to established guidelines.

Patients of both genders aged 18 years and older who underwent refractive surgery to correct their refractive errors were invited to participate. Patients with significant ocular conditions, such as keratoconus, corneal dystrophies, glaucoma, amblyopia, or severe dry eye, as well as those with a history of other ocular surgeries, were excluded. A convenience sampling method was used for recruitment, with invitations distributed via social media platforms and flyers posted at ophthalmic hospitals in Palestine.

Preoperative and postoperative measurements were taken by experienced optometrists at a six-month follow-up. These included best-corrected visual acuity (BCVA), UCVA, and the spherical equivalent (SE) of manifest refraction using a trial lens set. Visual acuity values were converted to the logarithm of the minimum angle of resolution (logMAR) units for statistical analysis. To avoid issues with statistical dependence between eyes, only right eye data were included in the analysis. The right eye was chosen based on the observation that the mean refractive and visual outcomes were comparable between both eyes in preliminary analysis. While this approach is commonly used in similar studies [5,11,12], we acknowledge that it may not capture inter-eye variability.

A structured questionnaire (see Appendices) was developed by the research team, informed by existing literature [5,11,12], to assess patient expectations, satisfaction, and QoL following refractive surgery. To ensure content validity, the questionnaire was pilot-tested on 15 patients who had previously undergone refractive surgery. Based on their feedback and review by two field experts, the questionnaire was refined for clarity and comprehensibility. The final version was used in the study, excluding data from the pilot participants.

Data were collected through structured telephone interviews conducted six months postoperatively by a trained researcher. Verbal informed consent was obtained prior to participation. The questionnaire comprised four main sections. The first collected demographic data, including age, gender, occupation, and educational level. The second addressed patients' motivations for undergoing surgery. The third section assessed overall satisfaction and happiness post-surgery as well as satisfaction with daily activities, such as reading, using digital devices, watching television (TV), playing sports, driving (day and night), shaving, applying makeup, and shopping. The final section evaluated common postoperative symptoms, including halos, glare, blurred vision, eye pain, discomfort, night vision difficulties, and dryness.

Patient satisfaction was rated using a 5-point Likert scale ranging from 0 (totally not satisfied) to 4 (totally satisfied), with higher scores indicating greater satisfaction. Participants who did not complete all questionnaire items were excluded from the final analysis.

This study was conducted in accordance with the principles of the Declaration of Helsinki. Ethical approval was obtained from the Institutional Review Board (IRB) of An-Najah National University (approval number: Med. Jan 2023/18). Informed consent was obtained from all participants before the study began.

IBM SPSS Statistics for Windows, Version 20.0 (SPSS Inc., Chicago, Illinois, United States), was used for data entry and statistical analysis. Based on a power analysis with 80% power, a two-sided significance level, and a medium effect size at an alpha level of 0.05, the study had sufficient power (≥80%) to evaluate satisfaction following refractive surgery, assuming a sample size of 100 participants. Statistical analyses were conducted using data from the right eyes of participants, as no significant difference was observed between the visual acuity of the right eye and the average of both eyes. Descriptive statistics were used to summarize the data. Continuous variables were expressed as means and standard deviations (mean±SD), while categorical variables were presented as frequencies and percentages. The Pearson correlation coefficient (r) was used to evaluate the strength and direction of linear relationships between continuous variables, including preoperative BCVA and postoperative UCVA, as well as between preoperative SE and overall satisfaction scores. Correlation strength was categorized as strong (r ≥ 0.6), moderate (0.3 ≤ r < 0.6), or weak (0.1 ≤ r < 0.3). Differences in satisfaction scores between males and females were assessed using the independent samples t-test. A p-value of ≤0.05 was considered statistically significant.

## Results

Demographic and clinical characteristics

The study included 100 patients, of whom 69% were female. The mean age of participants was 28.7±6.7 years, ranging from 20 to 47 years, with 43% falling within the 20-25-year age group. The mean preoperative SE was -2.4±0.8 diopters (D). In terms of refractive error, 72% of participants had myopia with astigmatism, 22% had spherical myopia, and 6% presented with hyperopia and astigmatism. A notable proportion of patients (56%) had a preoperative BCVA ranging from 1.1 to 1.5 logMAR. Regarding the type of refractive surgery performed, 40% underwent LASIK, 35% opted for PRK, and 25% received Femto SMILE surgery. Table 1 summarizes the demographics and clinical characteristics of the participants.

**Table 1:** Demographics and clinical characteristics of the participants. The data has been represented as percentages (%) (N=100) LASIK: laser in situ keratomileusis; PRK: photorefractive keratectomy; Femto SMILE: femtosecond-assisted small-incision lenticule extraction; BCVA: best-corrected visual acuity; SE: spherical equivalent

Characteristics	Percentage (%)
Gender	
Male	31%
Female	69%
Age	
20-25	43%
26-30	23%
31-35	16%
36-40	11%
40-45	7%
Occupation	
Employed	63%
Student	8%
Unemployed	29%
Education level	
Elementary school	1%
High school	18%
Diploma	4%
Bachelor degree	77%
Refractive error type	
Myopia only	22%
Myopia and astigmatism	72%
Hyperopia and astigmatism	6%
Surgery type	
PRK	35%
LASIK	40%
Femto SMILE	25%
Preoperative BCVA	
1.0 to 0.3	15%
0.2 to 0.0	29%
-0.1 to -0.2	56%
Preoperative SE	
+3.25 to +6.00	3%
+0.25 to +3.00	3%
-2.75 to plano	56%
-5.75 to -3.00	24%
-8.75 to -6.00	14%

Motivations for refractive surgery

The primary motivations for patients seeking vision correction through refractive surgery included reducing dependence on glasses (85%), improving visual acuity (80%), enhancing cosmetic appearance (76%), recommendation from previous patients (48%), work-related reasons (40%), difficulties with contact lens use (31%), the cost of spectacles and contact lenses (17%), participation in sports activities (14%), and frequent loss of glasses (8%).

Postoperative visual outcomes

The mean postoperative SE was -0.1±0.3 D, with a range from -0.25 to -1.00 D. Among the participants, 41% exhibited postoperative SE values ranging from -0.25 to -0.75 D, while 34% achieved full correction (plano). Additionally, 15% fell within the range of +0.25 to +0.75 D, 5% between -1.00 and -1.50 D, and another 5% between +1.00 and +1.50 D. Regarding postoperative UCVA, 86% of participants achieved values ranging from 0.0 to -0.2 logMAR, 12% ranged from 0.2 to 0.1 logMAR, and only 2% fell between 0.7 and 0.3 logMAR. A statistically significant moderate positive correlation was found between preoperative BCVA and postoperative UCVA (r=0.4; p<0.0).

Overall satisfaction and happiness

Ninety percent of patients expressed overall happiness following the refractive correction procedure (mean score: 3.9±0.3). Overall satisfaction was reported by 84% of patients (mean score: 3.8±0.3). Additionally, 93% indicated that they had achieved their primary goal (mean score: 3.9±0.2), and 97% expressed satisfaction with the elimination of glasses and contact lenses (mean score: 3.9±0.1). Seventy-five percent of patients reported achieving their expected visual acuity after the procedure (mean score: 3.7±0.5), and 82% expressed satisfaction with the speed of vision improvement (mean score: 3.8±0.4). Enhanced QoL was reported by 93% of participants (mean score: 3.9±0.2), and 96% indicated they would recommend the procedure to family or friends (mean score: 3.9±0.2). Males exhibited a significantly higher satisfaction score compared to females (t(98)=2.6; p=0.0), while no statistically significant association was found between age and satisfaction score (p>0.05). A strong and statistically significant positive correlation was found between overall satisfaction and preoperative SE (r=0.8; p<0.0), as well as between overall satisfaction and postoperative UCVA (r=0.6; p<0.0). Table 2 displays the detailed satisfaction scores, where a score of 4 signifies total satisfaction.

**Table 2:** The mean score (±SD) and the patient general satisfaction scale (%) post-operation. The data has been represented as percentages (%) (N=100) SD: standard deviation; CL: contact lenses

	Score	Totally satisfied	Satisfied	Neutral	Not satisfied	Totally not satisfied
Overall happiness	3.9±0.3	90%	10%	0%	0%	0%
							Overall satisfaction	3.8±0.3	84%	16%	0%	0%	0%
Explanation of procedure	3.6±0.7	77%	18%	2%	1%	2%							
							Expected visual acuity	3.7±0.5	75%	23%	1%	0%	1%
Elimination of glasses/CL	3.9±0.1	97%	3%	0%	0%	0%
							Goal achievement	3.9±0.2	93%	7%	0%	0%	0%
Satisfaction with vision improvement speed	3.8±0.4	82%	16%	2%	0%	0%
							Quality of life improvement	3.9±0.2	93%	7%	0%	0%	0%
Recommendation to friends/family	3.9±0.2	96%	3%	1%	0%	0%							

Satisfaction with daily activities

Participants were presented with more specific questions regarding their satisfaction with activities of daily living following the refractive correction procedure. Table 3 presents the results, indicating that 91% of patients reported an ability to read in daylight (mean score: 3.9±0.3), while 89% reported an ability to read under normal room illumination (mean score: 3.8±0.3). Postoperatively, 87% of participants indicated high satisfaction with mobile phone use (mean score: 3.8±0.3), while 69% reported slightly lower satisfaction with reading from a computer screen (mean score: 3.6±0.5). Participants reported a mean satisfaction of 3.9±0.3 for watching TV at home, with 92% expressing this level of satisfaction, while 72% reported a comparable score of 3.8±0.3 for watching movies in cinema theaters. Daylight driving was associated with a high satisfaction rate of 77% (mean score: 3.9±0.2), whereas night driving showed a slightly lower satisfaction level, with a mean score of 3.7±0.5, reported by 87% of participants. Over 75% reported high satisfaction with sports activities in general (mean score: 3.7±0.5) and swimming specifically (3.9±0.3). Additional activities, such as shaving, makeup application, and shopping, also demonstrated high satisfaction scores (mean scores: 3.8±0.4, 3.8±0.5, and 3.9±0.2, respectively). A statistically significant positive correlation was observed between overall satisfaction and all aspects of activities of daily living, including reading in daylight (r=0.5; p=0.0), mobile phone use (r=0.6; p<0.0), and night driving (r=0.4; p=0.0).

**Table 3:** The mean score (±SD) and the patient satisfaction scale for activities of daily living (%) post-operation. The data has been represented as percentages (%) (N=100) SD: standard deviation

	Score	Totally satisfied	Satisfied	Neutral	Not satisfied	Totally not satisfied
							Reading in daylight	3.9±0.3	91%	88%	1%	0%	0%
Mobile use	3.8±0.3	87%	13%	0%	0%	0%
Reading with room light	3.8±0.3	89%	99%	2%	0%	0%
Watching television	3.9±0.3	92%	7%	1%	0%	0%
Watching movies in the cinema	3.8±0.3	72%	28%	0%	0%	0%
Driving in daylight	3.9±0.2	77%	3%	20%	0%	0%
Driving at night	3.7±0.5	87%	12%	1%	0%	0%
Reading a computer screen	3.6±0.5	69%	27%	4%	0%	0%
							Sports activities	3.7±0.5	76%	20%	4%	0%	0%
Swimming	3.9±0.3	78%	0%	22%	0%	0%
Hair shaving	3.8±0.4	92%	6%	1%	1%	0%
Applying makeup	3.8±0.5	56%	9%	1%	1%	33%
Shopping	3.9±0.2	95%	5%	0%	0%	0%

Postoperative symptoms and dissatisfaction

The dissatisfaction rates among participants post-procedure were low, with reports indicating fewer than 10%, as illustrated in Table 4. The mean scores for various postoperative symptoms were as follows: dryness (1.4±1.3), glare and halos (0.7±1.0), eye pain (0.6±0.9), blurred vision (0.6±0.9), discomfort during activities of daily living (0.5±0.9), and night vision problems (0.5±0.8). No statistically significant correlations were observed between postoperative SE and symptoms of dryness (r=-0.0; p=0.4), glare and halos (r=0.0; p=0.6), eye pain (r=-0.1; p=0.3), blurred vision (r=0.0; p=0.6), discomfort during activities of daily living (r=-0.1; p=0.2), and night vision problems (r=0.0; p=0.7). Similarly, no significant correlations were found between UCVA and these symptoms: dryness (r=0.0; p=0.4), glare and halos (r=-0.0; p=0.7), eye pain (r=0.0; p=0.3), blurred vision (r=-0.0; p=0.5), discomfort during activities of daily living (r=0.1; p=0.3), and night vision problems (r=-0.0; p=0.6).

**Table 4:** The mean score (±SD) and the patient dissatisfaction scale of symptoms (%) post-operation. The data has been represented as percentages (%) (N=100) SD: standard deviation

	Score	Totally agree	Agree	Neutral	Disagree	Totally disagree
Dryness	1.4±1.3	8%	19%	14%	26%	33%
Glare and halos	0.7±1.0	2%	8%	7%	25%	58%
Eye pain	0.6±0.9	0%	5%	15%	20%	60%
Blurred vision	0.6±0.9	0%	7%	11%	17%	65%
Inability to perform daily activities	0.5±0.9	0%	6%	12%	10%	72%
							Night vision issues	0.5±0.8	0%	5%	8%	19%	68%

## Discussion

Assessing patient satisfaction following refractive surgeries is essential, as it represents a key indicator of the procedure's overall success [5,6,10-13]. Evaluation of surgical outcomes should extend beyond visual acuity and refractive correction, encompassing satisfaction, happiness, and perceived QoL postoperatively [5,12]. It is also important to assess the impact of the procedure on daily functioning and to identify potential sources of dissatisfaction from the patient's perspective [5,6,13]. This comprehensive approach aligns with SDG 3, which emphasizes the promotion of both physical and mental health. To the best of our knowledge, this is the first study in Palestine to assess the visual outcomes following refractive surgery while incorporating patient-reported satisfaction data. This contributes to the broader aim of SDG 10, which advocates for reducing inequalities in healthcare by generating locally patient-centered evidence.

Although the sample size in this study was relatively small, probably due to the high cost of refractive surgery and the tendency of individuals to pursue more affordable alternatives, it remains larger than that used in a previous study [11] and comparable to another [5]. The majority of participants were females (69%), reflecting a common trend among women in Middle Eastern countries to prioritize cosmetic concerns over the use of spectacles as a primary motivation for undergoing refractive surgery. This observation is consistent with findings from prior studies conducted in Palestine and other Middle Eastern contexts [7,13].

Postoperative visual outcomes in this study were favorable, with a mean SE of -0.1±0.3 D and 86% of patients achieving uncorrected visual acuity between 0.0 and -0.2 logMAR. These results align closely with previously reported findings [5,6,13]. In addition, 75% of participants reported total satisfaction with achieving their expected visual acuity, while 97% expressed total satisfaction with the elimination of spectacles and contact lenses following surgery, which is one of the most frequently cited motivators for undergoing refractive surgery in both this study and earlier research [6,10,11].

In this study, 84% of participants reported being satisfied with the outcomes of refractive surgery, with a mean satisfaction score of 3.8±0.3. Numerous previous studies have demonstrated favorable outcomes in terms of predictability, efficacy, and safety following corneal refractive surgeries [3,11-14], which have contributed to elevated patient expectations and high satisfaction rates ranging from 82% to 98% [3,11-14]. Additionally, 90% of participants reported a high level of overall happiness, with a mean score of 3.9±0.3 (4 indicates total satisfaction). Similarly, previous research has found that LASIK can significantly enhance patients' confidence, well-being, and overall happiness after surgery [14].

However, it is important to note that factors beyond the clinical success of the surgery may influence patient satisfaction and happiness, including inherent personality, age, and general physical health [12]. Evidence suggests that individuals who are naturally more optimistic, younger, or in better health may report high levels of satisfaction and happiness postoperatively [12].

In this study, 93% of patients reported an improvement in QoL following the surgery, with a mean score of 3.9±0.2. Satisfaction with the ability to perform daily activities, such as reading, driving, sports activities, and shopping, ranged from 56% to 95%. This section of the questionnaire was essential in evaluating the overall impact of refractive surgery, as clinical outcomes alone may not fully capture patient satisfaction without considering the individual's ability to perform everyday tasks [13]. Furthermore, 93% of participants indicated that they had achieved their primary goal, and 96% expressed willingness to recommend the procedure to others. These findings are consistent with those reported in previous studies [5,11,13].

Despite the high rate of patient satisfaction observed in this study, a small proportion of participants reported experiencing adverse symptoms following the refractive procedure. Specifically, 27% of patients reported symptoms of dry eye, a common postoperative complication [5,15] that is typically managed with artificial tears. Fewer than 10% of participants reported visual disturbances such as night vision problems, halos, glare, blurred vision, or eye pain. These findings are consistent with previous research [5,6,10,11,13], although the incidence of these adverse symptoms appears to be lower in the current study.

In most cases, such symptoms are transient and tend to resolve within several months postoperatively. For example, one study [6] reported that patients with high myopia experienced improved visual quality and fewer night vision complaints following surgery, with outcomes assessed at a mean follow-up of 24 months. It is also important to note that some patients may already experience such symptoms preoperatively while using corrective lenses, particularly in cases of myopia [16].

To obtain a more accurate assessment of the impact of refractive surgery on these symptoms, future studies should consider evaluating them both pre- and postoperatively. This would allow for a direct comparison and help differentiate between symptoms caused by surgery and those prior to the intervention. Additionally, a previous study [7] discovered that a combination of demographic, clinical, and lifestyle factors influenced patients' choices regarding vision correction methods. Refractive surgery, in particular, yielded the highest satisfaction rates among individuals who prioritized aesthetic outcomes and independence from daily use of corrective lenses [7].

While this study offers valuable insights into patient satisfaction and QoL after refractive surgery, several limitations should be noted. First, preoperative satisfaction levels were not assessed, which limits comparison with postoperative outcomes. Moreover, certain factors and potential confounders, such as physical health, income, or psychological well-being, that may influence happiness were not analyzed. In addition, the relatively small sample size, likely due to the high cost of surgery, limits the generalizability of the findings. The use of convenience sampling may also have introduced selection bias, as participants recruited via social media or hospital flyers may differ in motivation or satisfaction levels compared to the broader patient population.

Furthermore, the sample was predominantly myopic (94%), with only 6% hyperopic patients, which restricts applicability across different refractive error types. The unequal distribution among surgical techniques (40% LASIK, 35% PRK, and 25% Femto SMILE), combined with the limited sample size, further limited meaningful subgroup analysis. Additionally, most participants were young adults (66% aged 20-30), affecting the relevance of the results to older age groups. It is also important to note that satisfaction data were self-reported via telephone interviews, which may be subject to recall and interviewer bias and limit the standardization of responses. Finally, a six-month follow-up period may be too short to detect late-onset complications or assess long-term satisfaction outcomes.

Future research should consider enrolling a larger and more balanced sample regarding refractive error types, surgical techniques, and age groups to allow more robust comparisons. Longitudinal studies evaluating both preoperative and postoperative outcomes over extended follow-up periods (e.g., 1-2 years) would offer a more comprehensive understanding of long-term satisfaction and visual outcomes. Furthermore, future studies may benefit from incorporating objective outcome measures, such as in-person clinical assessments or digital self-administered surveys, to improve data reliability.

## Conclusions

This study demonstrated high levels of patient satisfaction, happiness, and improved QoL following refractive surgery in a Palestinian population. The majority of participants achieved excellent postoperative visual outcomes, with 86% attaining uncorrected visual acuity between 0.0 and -0.2 logMAR and a mean postoperative spherical equivalent of -0.1±0.3 D. A strong correlation was found between both preoperative refractive error and postoperative visual acuity with overall satisfaction scores. Furthermore, a significant proportion of patients reported improved ability to perform daily activities, such as reading, driving, and engaging in sports, with minimal adverse symptoms.

These findings highlight the effectiveness of refractive surgery not only in correcting visual impairments but also in enhancing overall patient well-being. As the first study of its kind in Palestine, it provides valuable insight into local patient outcomes and satisfaction, serving as a benchmark for future research and clinical practice. The results support the integration of patient-centered outcomes, such as satisfaction and QoL, into routine postoperative evaluations, ultimately guiding evidence-based decision-making and improving refractive surgery services within the region.

## Appendices

**Table 5:** Questionnaire

Questionnaire					
Do you agree to take part in this study? (Please select one option.)					
Yes					
No					
Gender					
Male					
Female					
Which of the following is your age group? (Please select one option.)					
20-25					
26-30					
31-35					
36-40					
40-45					
What is the highest level of education you have completed? (Please select one option.)					
Elementary school					
High school					
Diploma					
Bachelor's degree					
What is your current occupation? (Please select one option.)					
Employed					
Student					
Unemployed					
Other					
What were your reasons for undergoing refractive surgery?					
Please rate how important each reason was in your decision to have the surgery.					
(0=not important at all, 1=not important, 2=neutral, 3=important, 4=very important)					
	0	1	2	3	4
Freedom from spectacles					
Participation in sports or leisure activities					
Fear of losing or breaking spectacles					
Cosmetic appearance					
Expense of spectacles or contact lenses					
Improving career or work opportunities					
Please indicate your level of satisfaction with each of the following aspects related to your refractive surgery experience.					
(0=totally not satisfied, 1=not satisfied, 2=neutral, 3=satisfied, 4=totally satisfied)					
	0	1	2	3	4
Did you fully understand the procedure before treatment?					
Is your uncorrected sight as excellent as anticipated?					
Were you able to function after your eyes were treated?					
Are you currently wearing spectacles or contact lenses?					
How satisfied were you with the speed of improvement?					
Did you achieve the goals for which you had treatment?					
Has your quality of life improved?					
How happy are you, overall, that you had the treatment?					
Would you recommend the operation to a friend/family member?					
How satisfied are you with your ability to perform the following activities without spectacles or contact lenses after refractive surgery?					
(0=totally not satisfied, 1=not satisfied, 2=neutral, 3=satisfied, 4=totally satisfied)					
	0	1	2	3	4
Reading in daylight					
Mobile use					
Reading with room light					
Watching television					
Watching movies in the cinema					
Driving in daylight					
Driving at night					
Reading a computer screen					
Sports activities					
Swimming					
Hair shaving					
Applying makeup					
Shopping					
Please indicate your level of agreement with the following statements regarding symptoms you may have experienced after your refractive surgery.					
(0=totally agree, 1=agree, 2=neutral, 3=disagree, 4=totally disagree)					
	0	1	2	3	4
I have experienced dryness in my eyes.					
I have experienced glare and halos.					
I have experienced eye pain.					
I have experienced blurred vision.					
I have experienced discomfort during daily activities.					
I have experienced night vision issues.					
